# Effect of Feed Restriction during Pregnancy on Performance and Productivity of New Zealand White Rabbit Does

**DOI:** 10.4061/2011/839737

**Published:** 2011-09-04

**Authors:** Abeer Nafeaa, Souad Abd Elfattah Ahmed, Said Fat Hallah

**Affiliations:** ^1^Physiology Department, Faculty of Veterinary Medicine, Moshtohor, Benha University, Benha, Egypt; ^2^Animal Hygiene, Behavior and Management Department, Faculty of Veterinary Medicine, Moshtohor, Benha University, Benha, Egypt; ^3^Physiology Department, Faculty of Veterinary Medicine, Menofia University, El-Sadate Branch, El-Sadate City, Egypt

## Abstract

This study aimed to evaluate effect of stage of feed restriction on performance and productivity of pregnant does. New Zealand white female rabbits were randomly divided into three groups. Control group was provided daily with 185 g of food increased to 200 g from the 15th day of gestation. *R*_1_ was offered daily a restricted amount of food (60% restriction, 111 g) for the first half of pregnancy and then offered 200 g of food daily till parturition. *R*_2_ was provided with 185 g of food daily through the first half of pregnancy and then offered daily a restricted amount of food (60% restriction, 120 g) for the second half. After parturition, food was provided *adlibitum*. Maternal body weights, litter size, litter weight, and average body weight of kits at kindling of *R*_1_ showed no change, whereas *R*_2_ showed significant reduction in the weights of does at the 4th week of pregnancy and at kindling. The birth weight and weaning weight of *R*_2_ were significantly reduced. The highest mortality was recorded in kits of *R*_2_. No significant differences in blood parameters or serum prolactin were observed. The serum protein was significantly reduced *R*_2_.

## 1. Introduction

It is a common practice in commercial rabbit production to feed rabbit does to appetite directly after mating and during gestation. The young does fed *ad libitum* with diets of high energy level often show parturition problems, with the subsequent reduction of the number of newborn rabbits, linked to excessive fatness [1]. In order to reduce the excessive fatness of young does, restricted feeding during pregnancy is frequently applied to obtain uniformity in their body weights, to avoid fattening and high mortalities around parturition [2], to increase voluntary intake at the beginning of the lactation period, and to allow a long productive life [3]. Also, feed restriction could be exploited in the feeding regimen of rabbits, especially in periods of inadequate supply of concentrates and forages [4]. But, feed restriction for rabbits has to be considered as a stress condition and applied with attention when other stressors occur [5].

It is well known that fetal growth is dependent on an adequate supply of oxygen and nutrients crossing the placenta from the mother [6]. Moreover, nutrient supply to embryos and fetuses is exclusively dependent upon the mother [7]. Consequently, as the body size of the does decreases, the litter weight at kindling also reduces [8]. Furthermore, reduction of total caloric intake during pregnancy, eating disorders, and related indicators such as low pregnancy weights of does account for a significant proportion of intrauterine growth retardation [9]. Moreover, factors that perturb fetal substrate supply and are known to be responsible for much fetal growth restriction, such as placental insufficiency or poor maternal nutrition, are implicated in the long-term programming of adult dysfunction and disease [10]. Previous studies concluded that, after the restoration of maternal nutrition over the second half of gestation, fetal adipose tissue development is enhanced, which may act to place these individuals at increased risk of obesity in later life [11]. However, details of the influence of restricted feeding of the pregnant does on their blood parameters shortly after parturition and on the performance of their offspring are still obscure.

So, the aim of this work is to evaluate the effect of the stage of feed restriction during pregnancy on maternal body weights, weights of offspring at birth and weaning as well as some hematological and biochemical parameters of the young does.

## 2. Materials and Methods

### 2.1. Experimental Design

New Zealand white female rabbits (sixty females) of 20 weeks of age (first kindling) and weighed 2800 ± 100 g were randomly divided into three equal groups: *Group 1 (control group):* rabbits were provided daily with 185 g of food increased to 200 g from the 15th day of gestation (daily food intake of pregnant does according to [12]); *Group 2 *(*R*_1_ *, early feed restriction*)*:* rabbits were offered daily a restricted amount of food (60% restriction, 111 g) for the first half of pregnancy (the first fifteen days) and then offered 200 g of food daily till parturition; *Group 3 *(*R*_2_, * late feed restriction*) *:* rabbits were provided with 185 g of food daily through the first half of pregnancy and then offered daily a restricted amount of food (60% restriction, 120 g) for the second half of pregnancy. Treatments started after natural mating. After parturition, all does and their young were provided *ad libitum *amount of feed till weaning. Rabbits were reared in individual cages (50 cm × 50 cm × 30 cm) of galvanized wire net, equipped with an automatic drinker and a manual feeder. Rabbits were provided with a commercial pellet diet, containing 18% crude protein and 2700 Kcal/Kg ration metabolizable energy (ME). Does were submitted to a 16-hour photoperiod daily and the minimum ambient temperature was set at 18 ± 2°C. Fourteen days after mating, does were palpated to check pregnancy and nonpregnant does were excluded from the experiment. Five days before kindling, does were provided an access to nest boxes that were attached to each cage. Does were weighed individually at the start of the experiment then every two weeks and just after kindling. After kindling, the nest boxes were checked for live and still born kits. For each doe, the living kits were weighed to obtain the total litter weight at birth which were then divided by the number of kits (litter size at birth) to calculate the mean kits weight at birth. Then kits were weighed at 21 days of age and at the day of weaning (the 30th day). Kit's mortality rate % was recorded for each group from birth till weaning.

### 2.2. Blood Collection

Blood samples were collected from does five days after kindling [13]. The blood samples were collected in the morning (10.00 a.m.) from the ear veins. Two blood samples were taken from each female: one sample was taken into a tube containing EDTA for determination of the hematological parameters and the other was taken without an anticoagulant, centrifuged at 3000 g, and then the serum was collected and stored at −20°C until assayed for prolactin hormone and the total protein concentration.

### 2.3. Hematological and Biochemical Analysis

Packed cell volume (PCV), red blood cell number (RBCs), and hemoglobin concentration (Hb) were determined using Wintrobe's microhematocrite, improved Neubauer hemocytometer, and cyanomethemoglobin method, respectively. The erythrocytic indices, mean corpuscular volume (MCV), mean corpuscular hemoglobin (MCH), and mean corpuscular hemoglobin concentration (MCHC) were calculated according to [14]. Determination of serum prolactin was done according to [15] while determination of total protein concentration was done according to [16].

### 2.4. Statistical Analysis

Data collected were subjected to one-way ANOVA. Differences among means were tested by Duncan's multiple range test. 

## 3. Results and Discussion

Results of the effect of the stage of feed restriction during pregnancy on maternal body weights were presented in Table 1. Feed restriction during the first half of gestation did not affect the maternal body weights, whereas feed restriction during the second half of gestation was accompanied with significant reduction in the weights of does at the 4th week of pregnancy and at kindling. This decrease became nonsignificant from the second week after kindling till the end of the experiment. Similar results were recorded by many investigations which found that dams lost a significant amount of body weights throughout the gestation period as a result of undernutrition, but they were able to catch up to the *ad libitum *group by day 10 postnatal [17]. Other research recorded that, after delivery, the control group exhibited higher body weight than the restricted does, but at time of weaning all the mothers had similar live weights [13]. The results of the present study could be attributed to that as the postmiddle period of pregnancy is vulnerable to effects of reduced food consumption in pregnant rabbits [18], food-restricted females mobilized body fat and reduced their energy expenditure for maintenance and activity especially when feed restriction occurred shortly before parturition [19]. That could be the cause of the significant reduction observed in the weights of does subjected to feed restriction during the second half of gestation from the 4th week of pregnancy till the kindling. After kindling, these does were provided *ad libitum *amount of feed and they were able to catch up to the control group by the 15th day of delivery. That could be because, the animal under quantitative and qualitative feed restriction exhibited compensative growth as a consequence of increased food intake after restricted feeding [13]. Consequently, when food was provided *ad libitum *to the previously restricted does, weight gain was significantly higher than that in the *ad libitum *group [11, 20].

The present study revealed that feeding level during gestation period did not affect the litter size at birth (Table 2). There were no abortions, external, visceral, or skeletal malformations associated with any of the levels of maternal body weight loss due to feed restriction. Similar results were obtained in rabbits [21] and in rats [17]. However, previous studies investigated the effect of feeding level during first gestation in young does on reproductive performance and recorded a reduced number of live born kits as a result of low-feeding level during early gestation [22] and the litter size increased with increased weight of does [23, 24]. Also, feed restriction of pregnant does resulted in developmental abnormalities expressed by abortion, reduced fetal weight, and alterations in ossification [25]. One potential explanation for the results of the present study is that the young rabbit does could maintain normal blood supply to their embryos during this feeding restriction level as indicated by the hematological results which revealed no changes in hematological parameters due to feed restriction (Table 3).

In the present investigation it was clear that feed restriction during the first half of gestation increased the litter weight at birth and the mean kit weights from birth till weaning as compared to the control group but the differences were nonsignificant at any experimental periods (Table 2). These results disagree with the result obtained by a previous study which recorded a significant decrease in the weights of fetuses carried by does subjected to different degrees of feed restriction maintained for various periods during gestation [26, 27]. The obtained results could be ascertained by that, in the first and second weeks after feed restriction, compensatory feed intake occurred and does which ate more than average during the last week of gestation had heavier kits than does eating less than average feed intake [21], and in most instances, the energy level of the diet fed to the does influenced birth weight (higher energy level, greater weight) [2].

On the other hand, feed restriction during the second half of gestation significantly decreased the litter weight at birth as well as the postnatal pup weights; however, the differences were significant only from the 21th day till weaning (Table 2). Similar results were obtained by some previous studies which recorded that offsprings from undernourished group were significantly smaller at birth and were significantly less active at all ages independent of postnatal nutrition [28]. The reduced litter weight at birth of *R*_2_ in the present study could be attributed to the significantly reduced total protein concentration measured in the serum of the does (Table 3) where it is well known that the maternal body composition and diet are thought to affect the fetal development as a result of both direct effects on substrate availability to the fetus and indirectly through changes in placental functions and structure [29]. Furthermore, it was recorded that birth weight of rabbit kits was correlated with daily weight gain and weight at weaning [30]. So the reduction observed in mean kit weights of *R*_2_ from birth till weaning could be attributed to the reduced birth weight not to the amount of milk intake (no-significant differences were recorded between control and feed-restricted does in serum prolactin concentrations (Table 3).

As regarding the mortality rate % among the offspring from birth till weaning, the pups of feed-restricted does during the second half of gestation showed a significantly higher mortality rate % than the kits of the control does and feed-restricted does during the second half of gestation period (Table 2). The result disagreed with the result recorded in a previous research [13] which recorded similar number of rabbits at weaning as well as similar mortality rates for all groups (kits of control does and kits of does under different nutritive levels). The result recorded in the present study could be attributed to the significantly reduced litter weight (236.67 ± 8.82 g) as well as the reduced kit weight (48.22 ± 3.86 g) of *R*_2_ at birth, whereas within any given rabbit breed there is a close correlation between the weight of offspring at birth and their viability [18]. Furthermore, a previous study [31] concluded that, in the medium-sized breeds (including New Zealand white rabbits), the minimum birth weight consistent with the survival was between 40 and 45 g which was very close to the birth weight of *R*_2_ in the present study.

The blood parameters and serum prolactin concentrations of the does at the 5th day post partum were shown in Table 3; there were no significant differences in RBCs, Hb, PCV, MCV, MCH, or MCHC values between the control and feed-restricted groups. The obtained results agreed with the results obtained by [32] who concluded that hematological examination of pregnant does revealed variations in several blood parameters only in animals subjected to restricted feeding at 20 g/head/day when blood samples were collected on gestation day 19. Also, there were no significant differences recorded in serum prolactin concentrations between the control and feed-restricted groups. Similar results were obtained in rat [33], in ewes [11], and in mare [34], whereas, a previous study recorded some specific changes in the endocrine status during food restriction depending on the severity of food restriction and its duration [35].

Feed restriction during the first half of gestation did not affect the serum protein level whereas feed restriction during the second half of gestation significantly decreased the total protein as compared with the control group and this finding reflected low nutritive conditions due to restricted feeding as previously reported by [32, 36]. The significantly reduced total protein concentrations measured in serum of feed restricted does at the second half of gestation could be the cause of the significantly reduced weights of their litters at birth.

## 4. Conclusion

In order to reduce the excessive fatness of young rabbit does, restricted feeding during the first half (the first two weeks) of gestation is recommended as feed restriction for 15 days during early gestation does not affect the performance of young does, which had been fed to appetite during rearing.

## Figures and Tables

**Table 1:** The effect of the stage of feed restriction during pregnancy on maternal body weights (means ± SE).

Parameter	Group		
	Control	*R*_1_	*R*_2_
Weight of does at the start (g)	2860.00 ± 77.67^a^	2790.00 ± 165.63^a^	2873.33 ± 38.44^a^
Weight of does after two weeks (g)	3216.67 ± 72.65^a^	2966.67 ± 130.17^a^	3100.00 ± 76.38^a^
Weight of does after four weeks (g)	3500.00 ± 57.74^a^	3443.33 ± 34.80^a^	3196.67 ± 68.88^b^
Weight ofdoes at kindling day (g)	3140.00 ± 66.58^a^	3083.33 ± 95.28^a^	2783.33 ± 70.55^b^
Weight of does after six weeks (g)	3540.00 ± 95.39^a^	3490.00 ± 66.58^a^	3480.00 ± 58.59^a^
Weight of does after eight weeks (g)	3593.33 ± 63.59^a^	3556.67 ± 177.42^a^	3540.00 ± 63.51^a^

^a, b^Means with the different superscripts in the same raw are significantly different (*P* ≤ 0.05).

*R*_1_: early feed restriction.

*R*_2_: late feed restriction.

**Table 2:** The effect of the stage of feed restriction during pregnancy on litter performance (means ± SE).

Parameter	Group		
	Control	*R*_1_	*R*_2_
Litter Size at birth	6.00 ± 0.58^a^	5.67 ± 0.33^a^	5.00 ± 0.58^a^
Total litter weight at birth (g)	304.17 ± 8.21^a^	320.00 ± 17.32^a^	236.67 ± 8.82^b^
Mean kit weight at birth (g)	51.57 ± 4.60^a^	57.22 ± 6.55^a^	48.22 ± 3.86 ^a^
Mean kit weight at the 21st day (g)	380.00 ± 11.55^b^	446.67 ± 8.82^a^	308.33 ± 11.67^c^
Individual weaning body weight (g)	471.67 ± 14.81^b^	558.33 ± 16.19^a^	411.67 ± 7.26^c^
Mortality rate %	4.33 ± 0.67^a^	3.33 ± 0.88^a^	7.00 ± 0.58 ^b^

^a, b, c^ Means with the different superscripts in the same raw are significantly different (*P* ≤ 0.05).

*R*_1_: early feed restriction.

*R*_2_: late feed restriction.

**Table 3:** The effect of the stage of feed restriction during pregnancy on hematological findings of does at the 5th day post partum (means ± SE).

Parameter	Group		
	Control	*R*_1_	*R*_2_
RBCs (10^6^/*μ*l)	3.64 ± 0.27^a^	3.76 ± 0.32^a^	3.33 ± 0.15^a^
PCV (%)	30.65 ± 0.62^a^	29.26 ± 0.59^a^	28.34 ± 0.75^a^
Hb (g/dl)	9.14 ± 0.54^a^	9.24 ± 0.39^a^	8.31 ± 0.41^a^
MCV (fl)	84.79 ± 4.52^a^	78.61 ± 5.21^a^	85.39 ± 2.17^a^
MCH (pg)	25.15 ± 0.64^a^	24.74 ± 1.19^a^	24.98 ± 0.12^a^
MCHC (%)	29.79 ± 1.36^a^	31.59 ± 1.29^a^	29.30 ± 0.86^a^
Prolactin	3.85 ± 0.15^a^	4.20 ± 0.15^a^	3.51 ± 0.29^a^
Total protein (g/dl)	5.63 ± 0.15 ^a^	5.90 ± 0.26 ^a^	4.92 ± 0.16 ^b^

^a, b^Means with the different superscripts in the same raw are significantly different (*P* ≤ 0.05).

*R*_1_: early feed restriction.

*R*_2_: late feed restriction.
